# Impact of *CYP2C19*, *CYP3A4*, *ABCB1*, and *FMO3* genotypes on plasma voriconazole in Thai patients with invasive fungal infections

**DOI:** 10.1002/prp2.665

**Published:** 2020-10-30

**Authors:** Sumonrat Chuwongwattana, Thawinee Jantararoungtong, Santirat Prommas, Sadeep Medhasi, Apichaya Puangpetch, Chonlaphat Sukasem

**Affiliations:** ^1^ Division of Pharmacogenomics and Personalized Medicine Department of Pathology Faculty of Medicine Ramathibodi Hospital Mahidol University Bangkok Thailand; ^2^ Laboratory for Pharmacogenomics Somdech Phra Debaratana Medical Center (SDMC) Ramathibodi Hospital Bangkok Thailand; ^3^ Center for Medical Genomics Faculty of Medicine Ramathibodi Hospital Mahidol University Bangkok Thailand

**Keywords:** ABCB1, CYP2C19, CYP3A4, FMO3, invasive fungal infections, voriconazole

## Abstract

Voriconazole is the first‐line antifungal choice in the treatment of invasive fungal infections (IFIs). Single nucleotide polymorphisms (SNPs) in drug‐metabolizing and transporter genes may affect voriconazole pharmacokinetics. This study aimed to determine the frequency of the *CYP2C19* rs4244285, rs4986893, rs72552267, and rs12248560, *CYP3A4* rs4646437, *ABCB1* rs1045642, and *FMO3* rs2266782 alleles and determine the association between these genetic variants and voriconazole concentrations in Thai patients with invasive fungal infections. The study comprised 177 Thai patients with IFIs in whom seven SNPs in *CYP2C19*, *CYP3A4*, *ABCB1,* and *FMO3* were genotyped using *Taq*Man real‐time polymerase chain reaction (RT‐PCR) 5´ nuclease assays, and voriconazole plasma concentrations were measured by high‐performance liquid chromatography‐tandem mass spectrometry (LC‐MS/MS). Of the 177 patients included, 31 were <12 years and 146 were ≥12 years. The *CYP2C19* allele frequencies were 0.29 for *2, 0.060 for *3, 0.003 for *6, and 0.008 for *17. The allele frequency of *CYP3A4* (rs4646437) was 0.26, *ABCB1* (rs1045642) was 0.36, and *FMO3* (rs2266782) was 0.16. The median voriconazole dose/weight was significantly lower in patients aged ≥12 years when compared to the patients aged <12 years (*P* < .001). Patients aged <12 years with CYP2C19*1/*2 exhibited significantly higher median voriconazole plasma concentrations than those with the CYP2C19*1/*1 (*P* = .038). However, there were no significant differences in median voriconazole plasma concentrations among the *CYP2C19* genotypes in the patients aged ≥12 years. There was a lack of association observed among the *CYP3A4, ABCB1, and* FMO3 genotypes on the plasma voriconazole concentrations in both groups of patients. Our findings indicate that voriconazole plasma concentrations are affected by the CYP2C19*2 allele in patients aged <12 years but not in patients aged ≥12 years.

## Graphical abstract

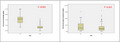

**Abbreviations**
IFIs: invasive fungal infections
LC‐MS/MS: liquid chromatography‐tandem mass spectrometry
RT‐PCR: real‐time polymerase chain reaction
SNPs: Single nucleotide polymorphisms

## 1 INTRODUCTION

Voriconazole is a second‐generation triazole with broad‐spectrum spectrum antifungal activity against *Aspergillus*, fluconazole‐resistant *Candida*, *Fusarium,* and *Scedosporium* species. It is used as a first‐line treatment for invasive fungal infections (IFIs).^1^ Voriconazole is characterized by a narrow therapeutic range, significant inter‐ and intrapatient pharmacokinetic variability, and nonlinear pharmacokinetics.^2^ Voriconazole exhibits significant exposure‐response relationships for efficacy and toxicity, which is why therapeutic drug monitoring (TDM) is now widely recommended to optimize clinical outcomes.^3^ Previous studies have reported an optimal voriconazole concentration of 1.0‐5.5 µg/mL for improved outcomes and minimal toxicities.^4^

Voriconazole is metabolized by the hepatic cytochrome P450 isoenzymes, CYP2C19, CYP2C9, and CYP3A4.^5^ Flavin‐containing monooxygenase 3 (FMO3), an oxidative drug‐metabolizing enzyme, also contributes to the metabolism of voriconazole.^6^ *CYP2C19* polymorphism, age of the individual, liver function, and coadministration of drugs that modulate its activities may affect voriconazole plasma concentrations.^7^ Genetic variations in the metabolizing enzyme CYP2C19 are one of the major determinants of the voriconazole blood level. The Clinical Pharmacogenetics Implementation Consortium (CPIC) has provided therapeutic recommendations for the use of voriconazole based on *CYP2C19* genotype for pediatric and adult patients.^8^ Two *CYP2C19* alleles, rs4244285 (CYP2C19*2, c.681G > A) and rs4986893 (CYP2C19*3, c.636G > A), decrease the enzyme activity by the quantitative reduction of enzyme protein and have been reported to result in approximately fourfold higher plasma exposure to voriconazole than CYP2C19*1 carriers. The CYP2C19*17 (c.‐806C > T, rs12248560) allele is associated with increased enzyme activity and has been associated with therapeutic failure with voriconazole.^9^, ^10^ The CYP2C19*6 (c.395G > A, rs72552267) is a no function allele with frequency <0.5% in major ethnic groups.^11^ CYP3A4 enzyme is the most abundant in human liver and plays an important role in oxidative metabolism of clinical drugs in adult liver.^12^ Voriconazole is catalyzed more efficiently by CYP3A4 than CYP3A5.^13^ The rs4646437 single‐nucleotide polymorphism (SNP) of the *CYP3A4* was associated with higher plasma voriconazole concentrations in a study cohort of Chinese patients with IFIs.^14^ Allegra et al reported an association between the *ABCB1* (c.3435C > T, rs1045642) SNP and trough concentration of voriconazole.^15^ The study found that the genotype TT was associated with a decreased trough concentration of voriconazole in children as compared to genotypes CC and CT. FMOs, mainly the FMO3 enzyme, have an important role in voriconazole metabolism that have been reported in the several studies, especially among children.^6^, ^16^ In vitro study suggested a contribution of approximately 25% of FMO3 enzyme might affect voriconazole metabolism.^16^ To our knowledge, the influence of *FMO3* genetic polymorphisms on the pharmacokinetic parameters of voriconazole in invasive fungal infection patients remains unclear. *FMO3* rs2266782 *(E158K)* showed decreased activity. This variation was reported in multiple populations, including Caucasians, Asians and African‐Americans.^17^ Yamada et al reported an association between *FMO3* rs2266782 *(E158K)* and trough concentration of voriconazole.^9^

Based on these observations, we sought to determine the frequency of the *CYP2C19* rs4244285, rs4986893, rs72552267, and rs12248560, *CYP3A4* rs4646437, *ABCB1* rs1045642, and *FMO3* rs2266782 polymorphisms in a Thai population and to determine the association between voriconazole plasma concentrations and these genetic variants in Thai patients with invasive fungal infections.

## 2 MATERIALS AND METHODS

### 2.1 Patients

This was a retrospective study of 177 Thai invasive fungal‐infected patients receiving voriconazole treatment from January 2013 to December 2015 at Ramathibodi Hospital (Bangkok, Thailand). All patients were started on standard voriconazole dosing either oral or intravenous administration every 12 hours. For intravenous (IV) dose, patients received voriconazole at a loading dose of 6 or 8 mg/kg/dose twice on the first day of treatment, then 4 or 6 mg/kg/dose twice daily. For oral (PO) dose, the recommended dose of voriconazole was 100 mg twice daily for a patient with body weights less than 40 kg and 200 mg twice daily for more than 40 kg.

Inclusion criteria included all of the following: diagnosis of invasive fungal infection, treatment with voriconazole, and with at least one steady‐state voriconazole trough concentration. Exclusion criteria were as follows: current pregnancy, allergy to voriconazole, and ethnicity other than Thai. Patient medical records were individually reviewed using a standardized data collection template. Collected data included demographic information and clinical information regarding the voriconazole dosing and delivery route, concomitant medications taken during voriconazole therapy, treatment response, and adverse events.

The study protocol was approved by the Hospital Ethics Committee of Ramathibodi Hospital, Mahidol University. All patients provided written informed consent. This study is registered in the Voriconazole Registry‐Prospective cohort.

### 2.2 Genotyping assay

Genomic DNA was extracted from EDTA blood sample using a MagNA Pure LC DNA Isolation Kit following the manufacturer's protocol (Roche, Mannheim, Germany). Genomic DNA was quantified using NanoDrop ND‐1000 Spectrophotometer (Thermo Fisher Scientific, DE, USA). Genomic DNA was then frozen at −20°C until the time of genotyping.

Genotyping was performed by allelic discrimination using *Taq*Man real‐time polymerase chain reaction (RT‐PCR) 5′ nuclease assays. Allelic discrimination was carried out by measuring endpoint fluorescence intensity with a ViiA™ 7 RT‐PCR System (Applied Biosystems, Foster City, California).

### 2.3 Blood sampling and analytical assays

Blood samples were taken in steady‐state one hour before voriconazole administration. Voriconazole trough plasma concentrations were determined using validated high‐performance liquid chromatography‐tandem mass spectrometry (LC‐MS/MS).^18^ All intra‐ and interday accuracy (%) and precision (%CV) measurements were within acceptable ranges recommended by the US‐FDA guidelines for bioanalytical method validation.^19^ The lower limit of quantification (LLOQ) for voriconazole was 0.05 µg/mL. The intra‐ and interday accuracies were 95.30 ± 3.41% and 87.80 ± 6.58%, respectively. The intra‐ and interday precision (%CV) were 7.2% and 7.9%, respectively.

### 2.4 Statistical analysis

Continuous data are presented as mean and standard deviations, and categorical data are expressed as frequencies and percentages. Conformity to Hardy‐Weinberg equilibrium (HWE) was determined using Fisher's exact test. Kruskal‐Wallis and Mann‐Whitney tests were used to determine the influence of genetic polymorphisms on voriconazole plasma concentrations, and the correlation of age with dose and plasma concentrations of voriconazole. A two‐sided *P*‐value less than .05 was considered statistically significant. Statistical analyses were performed using the SPSS software package (SPSS version 18.0 for Windows, SPSS Inc, Chicago, IL).

## 3 RESULTS

### 3.1 Patient characteristics

The demographics and clinical characteristics of recruited patients (n = 177) are summarized in Table 1. Patients were categorized based on the age group (age <12 years, age ≥12 years). Thirty‐one patients were aged <12 years, and 146 were ≥12 years. The mean (SD) age of the patients aged <12 years was 5.00 years (2.93 years) and ≥12 years was 48.82 years (21.01 years). The mean (SD) body weight in patients aged <12 years was 21.36 kg (11.24 kg) and ≥12 years was 55.83 kg (11.28 kg). Most of the patients had hematological disorders (<12 years, n = 22 (70.97%) and ≥12 years, n = 73 (50%)). The median dose (mg/kg) of voriconazole was 7.894 mg/kg in patients aged <12 years and 4 mg/kg in ≥12 years. Voriconazole was used by 96.77% of patients aged <12 years for possible invasive pulmonary aspergillosis (IPA). Meanwhile, only 19.86% of patients aged ≥12 years used voriconazole for possible IPA, 47.26% for probable IPA, and 28.08% for proven IPA. Although, voriconazole was also prescribed for 4.80% of patients aged <12 years with no IPA. Seventeen (54.84%) patients received voriconazole intravenously and 14 (45.16%) orally in patients aged <12 years. In patients aged ≥12 years, 116 (79.45%) received voriconazole orally and 30 (20.55%) intravenously.

**TABLE 1:** Baseline characteristics of the patients categorized by age (n = 177)

Characteristics	Number	
	Age < 12 years (N = 31)	Age ≥ 12 years (N = 146)
Sex		
Male	23 (74.19%)	66 (45.21%)
Female	8 (25.81%)	80 (54.79%)
Age	5.00 ± 2.93	48.82 ± 21.01
Weight (Mean ± SD)	21.36 ± 11.24	55.83 ± 11.28
Underlying disease		
Hematological disorder	22 (70.97%)	73 (50.00%)
Lymphoma	2 (6.45%)	19 (13.01%)
Diabetes	0 (0)	12 (8.22%)
SLE	0 (0)	11 (7.53%)
Thalassemia	0 (0)	3 (2.05%)
Malignant	4 (12.90%)	0 (0)
Cancer	0 (0)	2 (1.37%)
Hypertension	0 (0)	2 (1.37%)
Others	2 (6.45%)	19 (13.01%)
None	1 (3.23%)	5 (3.42%)
Drug dose/body weight (Median (IQR)), mg/kg	7.894 (5.970‐9.000)	4.000 (3.633‐4.545)
Type of IPA		
Possible IPA	30 (96.77%)	29 (19.86%)
Probable IPA	1 (3.23%)	69 (47.26%)
Proven IPA	0 (0)	41 (28.08%)
No IPA	0 (0)	7 (4.80%)
Route of administration		
Intravenous	17 (54.84%)	30 (20.55%)
Oral	14 (45.16%)	116 (79.45%)
Infection site		
Pulmonary	31 (100%)	118 (80.82%)
Extra pulmonary	0 (0)	28 (19.18%)

### 3.2 Allelic frequency of the variants in a Thai population treated with voriconazole

The frequencies of the variants in *CYP2C19*, *CYP3A4*, *ABCB1*, and *FMO3* are shown in Table 2. *CYP2C19* allele frequencies were 0.29 for *2, 0.060 for *3, 0.003 for *6, and 0.008 for *17. The allele frequency of *CYP3A4* (rs4646437) was 0.26, *ABCB1* (rs1045642) was 0.36, and *FMO3* (rs2266782) was 0.16. The allele frequencies of all the variants were in Hardy‐Weinberg equilibrium (*P* > .05).

**TABLE 2:** The minor allele frequencies of pharmacokinetics and transporter in Thai patients with invasive fungal infections (n = 177)

Gene	rs number	Polymorphisms	Genotype	No. (%)	Minor allele frequency
CYP2C19*2	rs4244285	*681G > A*	GG	88 (49.72)	*A* = 0.290
			GA	75 (42.37)	
			AA	14 (7.91)	
CYP2C19*3	rs4986893	*636G > A*	GG	156 (88.14)	*A* = 0.060
			GA	21 (11.86)	
CYP2C1*6	rs72552267	*395G > A*	GG	176 (99.44)	*A* = 0.003
			GA	1 (0.57)	
CYP2C19*17	rs12248560	*−806C > T*	CC	174 (98.31)	*T* = 0.008
			CT	3 (1.69)	
*CYP3A4*	rs4646437	*671‐202C > T*	CC	90 (66.10)	*T* = 0.26
			CT	81 (30.51)	
			TT	6 (3.39)	
*ABCB1*	rs1045642	*3435C > T*	CC	75 (42.37)	*T* = 0.36
			CT	78 (44.07)	
			TT	24 (13.56)	
*FMO3*	rs2266782	*G > A*	GG	129 (72.89)	*A* = 0.16
			GA	41 (23.16)	
			AA	7 (3.95)	

### 3.3 Influence of CYP2C19 genotypes on plasma voriconazole concentrations

The association of *CYP2C19* polymorphisms with voriconazole dose and voriconazole plasma concentrations in Thai patients with invasive fungal infections is shown in Table 3. There was considerable interindividual variability in voriconazole trough concentrations among the patients aged <12, which ranged from 1.13 μg/mL to 5.13 μg/mL. Patients aged <12 years with CYP2C19*1/*2 exhibited significantly higher median voriconazole plasma concentrations than those with the CYP2C19*1/*1 (*P* = .038). CYP2C19*2/*2 was not associated with increased trough levels versus CYP2C19*1/*1 (*P* = .312) among the patients aged <12 years. CYP2C19*17 allele was not detected among the patients aged <12 years. No significant differences were observed in median voriconazole doses among the *CYP2C19* genotypes in the patients aged <12 years.

**TABLE 3:** Median initial voriconazole dose and voriconazole trough concentration (μg/mL) stratified by *CYP2C19* polymorphisms in Thai patients with invasive fungal infections (n = 177)

Gene polymorphism	Frequency (%)	Median voriconazole dose (mg/kg) (IQR)	*P*‐value^a^	Median Voriconazole level (μg/mL) (IQR)	*P*‐value^a^
Patients < 12 years					
*CYP2C19* * *1/* * *1*	12 (38.71)	7.98 (6.73‐9.29)	Ref.	1.130 (0.516‐3.894)	Ref.
*CYP2C19* * *1/* * *2*	12 (38.71)	7.61 (5.93‐9.71)	1.000	4.271 (2.066‐7.716)	0.038*
*CYP2C19* * *1/* * *3*	3 (9.68)	7.89 (7.52‐8.58)	0.885	5.125 (2.959‐6.040)	0.312
*CYP2C19* * *2/* * *2*	3 (9.68)	7.14 (6.30‐7.66)	0.427	3.793 (2.436‐4.008)	0.386
*CYP2C19* * *2/* * *3*	1 (3.23)	4.69	0.109	2.523	0.593
	31 (100.00)	7.89 (5.97‐9.00)	0.517	2.726 (0.898‐6.956)	0.288
Patients ≥ 12 years					
*CYP2C19* * *1/* * *1*	59 (40.41)	3.80 (3.43‐4.56)	Ref.	2.020 (0.236‐0.856)	Ref.
*CYP2C19* * *1/* * *2*	55 (37.67)	4.00 (3.70‐4.44)	0.223	2.340 (0.205‐0.980)	0.244
*CYP2C19* * *1/* * *3*	12 (8.22)	4.04 (3.89‐4.36)	0.315	2.380 (0.117‐1.606)	0.524
*CYP2C19* * *2/* * *17*	1 (0.68)	7.55	0.088	0.604	0.388
*CYP2C19* * *2/* * *2*	11 (7.53)	4.22 (3.84‐4.68)	0.188	2.703 (0.213‐1.116)	0.540
*CYP2C19* * *2/* * *3*	5 (3.42)	3.80 (3.66‐4.17)	0.793	2.585 (0.252‐1.340)	0.613
*CYP2C19* * *2/* * *6*	1 (0.68)	2.99	0.194	2.520	0.260
*CYP2C19* * *1/* * *17*	2 (1.37)	4.03 (3.39‐4.67)	0.903	2.665 (0.250‐1.227)	0.624
	146 (100.00)	4.00 (3.63‐4.54)	0.327	2.105 (0.870‐3.887)	0.864

Note

Ref., Reference (*CYP2C19* * *1/* * *1*, wild type).

^a^ *P*‐value was calculated using Kruskal‐Wallis and Mann‐Whitney U test. Comparison with CYP2C19 *1/*1.

* *P*‐value < *0.05*.

There were no significant differences in median voriconazole plasma concentrations among the *CYP2C19* genotypes in the patients aged ≥12 years. The median voriconazole level ranged from 0.60 μg/mL to 2.7 μg/mL in the patients aged ≥12 years. In patients aged ≥12 years, only one patient had CYP2C19*2/*17 genotype and the trough concentration out of therapeutic range (<1 µg/mL). No significant differences were observed in median voriconazole doses among the *CYP2C19* genotypes in the patients aged ≥12 years. The median voriconazole dose/weight was almost double in patients aged <12 years compared to patients aged ≥12 years (7.89 mg/kg vs 4.00 mg/kg).

### 3.4 Influence of CYP3A4, ABCB1, and FMO3 genotypes on plasma voriconazole concentrations

There was a lack of association observed among the *CYP3A4, ABCB1, and* FMO3 genotypes on the plasma voriconazole concentrations in both groups of patients as shown in Table 4. In patients aged <12 years, *CYP3A4* rs4646437 CT (n = 16) genotype showed a median voriconazole level of 4.363 μg/mL (IQR: 1.082‐7.510 μg/mL) which was relatively higher compared to CC (n = 14) and TT (n = 1) genotypes. Although not statistically significant, the median voriconazole dose was relatively higher in the *CYP3A4* rs4646437 CT genotype among the patients aged <12 years (Table 4). *FMO3* rs2266782 AA genotype was not observed among the patients aged <12 years.

**TABLE 4:** Association between genetic polymorphisms with voriconazole dose (mg/kg) and initial voriconazole trough concentration (μg/mL) in Thai patients with invasive fungal infections (n = 177)

Gene polymorphism	Frequency (%)	Median voriconazole dose mg/kg (IQR)	*P*‐value^a^	Median Voriconazole level (μg/mL) (IQR)	*P*‐value^a^
Patients < 12 years (N = 31)					
*CYP3A4(rs4646437)*					
*CC*	14 (45.16)	6.72 (5.85‐8.15)	Ref.	1.633 (0.989‐3.406)	Ref.
*CT*	16 (51.61)	8.03 (7.04‐8.78)	0.178	4.363 (1.082‐7.510)	0.125
*TT*	1 (3.23)	4.09	0.108	2.406	0.789
*ABCB1(rs1045642)*					
*3435C > T*					
*CC*	12 (38.71)	7.14 (6.14‐8.03)	Ref.	3.793 (1.403‐5.729)	Ref.
*CT*	14 (45.16)	7.14 (5.97‐8.28)	0.401	1.600 (0.898‐4.223)	0.977
*TT*	5 (16.13)	8.33 (7.14‐9.00)	0.571	5.063 (2.773‐6.956)	0.364
*FMO3* (rs2266782)					
*G > A*					
*GG*	24 (77.42)	7.51 (5.97‐8.28)	Ref.	2.464 (0.794‐4.503)	Ref.
*GA*	7 (22.58)	7.14 (6.30‐8.66)	0.919	5.063 (2.651‐8.861)	0.133
Patients ≥ 12 years (N = 146)					
*CYP3A4(rs4646437)*					
*CC*	76 (52.05)	3.90 (3.63‐4.54)	Ref.	2.105 (0.960‐3.533)	Ref.
*CT*	65 (44.52)	3.93 (3.43‐4.44)	0.480	2.440 (0.750‐4.690)	0.630
*TT*	5 (3.43)	4.54 (4.21‐4.65)	0.232	1.900 (1.350‐2.340)	0.878
*ABCB1(rs1045642)*					
*3435C > T*					
*CC*	63 (43.15)	3.85 (3.57‐4.70)	Ref.	1.563 (0.605‐3.795)	Ref.
*CT*	64 (43.83)	4.00 (3.63‐4.44)	0.919	2.320 (1.256‐3.843)	0.250
*TT*	19 (13.02)	3.89 (3.38‐4.28)	0.436	1.293 (0.475‐3.037)	0.676
*FMO3*(rs2266782)					
*G > A*					
*GG*	105 (71.92)	4.00 (3.63‐4.45)	Ref.	2.330 (0.915‐3.730)	Ref.
*GA*	34 (23.29)	3.91 (3.65‐4.54)	0.962	1.563 (0.750‐4.160)	0.525
*AA*	7 (4.79)	3.48 (3.09‐4.10)	0.133	2.720 (0.970‐5.530)	0.762

Note

Ref., Reference (wild type).

^a^ *P*‐value was calculated using Kruskal‐Wallis and Mann‐Whitney U test. Comparison with *GG* or *CC*.

### 3.5 Relationship of age with dose and plasma concentrations of voriconazole

**FIGURE 1:**
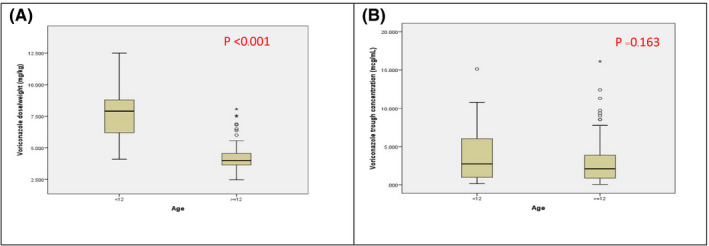
Association between (A) voriconazole dose (mg/kg) (B) initial voriconazole trough concentration (µg/mL) and age group in Thai patients with invasive fungal infections (n = 177)

Figure 1 shows the relationships of the age with the dose and the trough plasma concentrations of voriconazole. The median voriconazole doses in patients aged <12 years and patients aged ≥12 years were 7.89 mg/kg (IQR: 5.97‐9.00 mg/kg) and 4.00 mg/kg (IQR: 3.63‐4.54 mg/kg), respectively. The median voriconazole dose/weight was significantly lower in patients aged ≥12 years when compared to the patients aged <12 years (*P* < .001). The median voriconazole trough concentrations in patients aged <12 years and patients aged ≥12 years were 2.726 μg/mL (IQR: 0.898‐6.956 μg/mL) and 2.105 μg/mL (0.870‐3.887 μg/mL), respectively. There was no statistically significant difference in plasma voriconazole concentrations between the two age groups (*P* = .163).

## 4 DISCUSSION

In this study, we investigated the association of SNPs in *CYP2C19*, *CYP3A4*, *ABCB1*, and *FMO3* and the plasma concentrations of voriconazole in Thai patients with invasive fungal infections. Also, the allele frequency of these variants in the Thai population was obtained. The results demonstrated that *CYP2C19* rs4244285 was associated with increased plasma concentrations of voriconazole in *1/*2 genotype when compared to the wild type *1/*1 genotype in patients aged <12 years. This finding indicates that rs4244285 may be accountable for the susceptibility to changes in the plasma voriconazole concentrations. To our knowledge, this report is the first to analyze the involvement of *CYP2C19*, *CYP3A4*, *FMO3*, and *ABCB1* variants that affects the plasma concentrations of voriconazole in Thai patients.

Voriconazole, an approved treatment for IFIs, exhibits nonlinear pharmacokinetics and is primarily metabolized by hepatic cytochrome P450 (CYP) enzymes 2C19 (CYP2C19) and CYP3A4. Genetic polymorphisms in *CYP2C19* play an important role in the wide interindividual variability in voriconazole pharmacokinetics.^20^ In a population of 106 South‐western Chinese Han patients, voriconazole dose‐corrected predose concentration (C_0_/dose) was significantly influenced by the *CYP2C19* genotype. The voriconazole C_0_/dose was significantly associated with the CYP2C19*2 (rs4244285) polymorphism.^21^ CYP2C19*1 represents the wild type allele.^22^ The CYP2C19*2 (c.681G > A; rs4244285) loss‐of‐function allele is responsible for the *CYP2C19* “poor‐metabolism” phenotype. The CYP2C19*2 allele, a synonymous G > A transition in exon 5, creates an aberrant splice site and alters the mRNA reading frame, which results in a truncated, nonfunctional protein.^23^ The frequencies of CYP2C19*2 are approximately 12% in Caucasians, 15% in African‐Americans, and 29%‐35% in Asians.^24^ The frequency of CYP2C19*2 in our study was found to be 29% and agrees with the data published earlier by Sukasem et al.^25^ We found differences in voriconazole plasma concentrations in heterozygous carriers of the CYP2C19*2 allele compared to noncarriers in patients aged <12 years. However, the same association was not found in patients aged ≥12 years. This supports the voriconazole pharmacokinetic variability between children and adults previously addressed by Neely et al.^26^ Contradictory results were recently reported in elderly patients. The authors reported that the median voriconazole concentration in patients with the CYP2C19*1/*2 and CYP2C19*2/*2 genotypes were significantly higher than those in patients with the CYP2C19*1/*1 genotype.^27^, ^28^ The voriconazole C0/dose was significantly associated with the CYP2C19*2 (rs4244285) polymorphism especially in patients aged <12 years. One possible reason is probably because the relative contribution of CYP2C19 enzymes in the formation of voriconazole N‐oxide is 50% and 35% in children and adults, respectively.^6^

Both CYP2C19*3 and CYP2C19*6 alleles included in our study are associated with reduced capability for metabolizing drugs. The frequencies of the CYP2C19*3 have a higher prevalence in Asian populations ranging from 2% to 9% compared to other major ethnic populations. In this study, we found that the incidence of CYP2C19*3 in Thai population (0.060) was similar to that found in East Asian populations (0.089).^23^ The frequencies of the CYP2C19*6 allele in most populations are below 1% which was evident in our study population with a frequency of 0.3%. The CYP2C19*17 allele is a −806 C > T SNP that creates a consensus binding site for the GATA transcription factor family, resulting in increased gene transcription and high enzyme activities.^23^ The CYP2C19*17 allele was observed with a prevalence of 0.8% among the Thai population in this study, which is lower than the previously reported frequency in the African (16%), American (18%), East Asian (2.7%), and European (21%) populations.^24^

In Thai population, the *FMO3* allele frequency was not reported. In our study, the minor allele frequency of *FMO3* rs2266782 *(E158K)* in Thais was 0.160 that showed a lower frequency than in other ethnic populations.^17^ Yamada et al reported an association between *FMO3* rs2266782 *(E158K)* and lower plasma concentration of voriconazole.^9^ In contrast, this study was not associated with the plasma concentration of voriconazole. In a previous study, He et al reported the association between *CYP3A4* rs4646437 and higher plasma concentrations of voriconazole in Chinese.^14^, ^29^ In this study, the minor allele frequency of *CYP3A4* rs4646437 was different from other ethnicities. The minor allele frequency in Thai population was higher than either that of Asians or Caucasians (0.26, 0.12 and 0.08 respectively).^29^ In contrast, in this study we did not find any statistically significant association between *CYP3A4* rs4646437 and plasma voriconazole concentrations.

To our knowledge, numerous variants of *CYP3A4* have been described, but none of them can be held accountable for the high phenotypic variation observed.^30^ In addition to *CYP3A5*, CYP3A5*3 is the major of *CYP3A5* allele that found in all ethnic groups.^31^, ^32^ To date, SNPs in *CYP3A4* and *CYP3A5* seem of limited importance to voriconazole.^14^, ^33^ Weiss et al found no correlation between the CYP3A5*3 and voriconazole pharmacokinetics and Levin et al found that CYP3A5*3 was not associated with the liver toxicity that was caused due to voriconazole treatment.^33^, ^34^ P‐glycoprotein (P‐gp) is encoded by the *ABCB1* gene that is the most important drug efflux transporter and has been associated with voriconazole pharmacokinetics. Several SNPs have been reported in *ABCB1* related to altered transport function of P‐gp leading to interindividual variability in the pharmacokinetics of its substrates. In this study, the minor allele frequency of *ABCB1*rs1045642 *(C3435T)* in Thai was 0.36 that was lower than previously reported from other studies on the Thai population.^35^

Most studies have focused on *CYP2C19* and voriconazole disposition. We studied the polymorphisms in *CYP3A4*, *FMO3*, and *ABCB1* and their impact on plasma voriconazole levels. We did not observe any significant association between these gene variants and plasma voriconazole levels in the patients aged <12 years and ≥12 years.

Several studies have demonstrated the relationship between treatment response and adverse events of voriconazole and its plasma concentrations.^10^, ^36^, ^37^ The currently recommended voriconazole plasma level ranges for improved outcomes and minimized toxicities are in the range of 1.0‐5.5 or 1.0‐6.0 μg/mL.^38^ Trough concentrations 1.0‐6.0 μg/mL (within therapeutic range) were observed in 99% of the patients in our study. Considering the age effect on voriconazole dosing, pediatric patients need higher loading and maintenance dosing of voriconazole than in adult patients due to enhanced hepatic clearance and first‐pass effect.^39^ We found that the median voriconazole dose/weight was significantly lower in patients aged ≥12 years when compared to the patients aged <12 years (Figure 1).

Our study has some limitations. First, it was a retrospective and single‐center study. Second, a small number of patients were included. Consequently, the CYP2C19*6 and *17 allele variants were absent in patients aged <12 years. Also, some homozygous mutations did not appear. Third, the focus of our study was on the influence of genetic polymorphisms in the *CYP2C19*, *CYP3A4*, *FMO3*, and *ABCB1* genes (CYP2C19*2*3*6 and *17 CYP3A4 rs464643*7, FMO3* rs2266782 and *ABCB1* rs1045642*)* on voriconazole plasma concentrations, but the clinical outcome was not evaluated. Further studies are required to describe other clinically important SNPs of *CYP2C19, CYP3A4, CYP3A5* and *FMO3*.

In conclusion, we report the frequency of *CYP2C19* rs4244285, rs4986893, rs72552267, and rs12248560, *CYP3A4* rs4646437, *ABCB1* rs1045642, and *FMO3* rs2266782 alleles in a Thai population and the involvement of these variants in the voriconazole plasma concentrations. Our data might support individual dose adjustment of voriconazole according to genetic polymorphisms of *CYP2C19*.

## 5 Ethics statement

The study protocol was approved by the Hospital Ethics Committee of Ramathibodi Hospital, Mahidol University. All patients provided written informed consent. This study is registered in the Voriconazole Registry‐Prospective cohort.

## CONFLICT OF INTEREST

The authors declare that the research was conducted in the absence of any commercial or financial relationships that could be construed as a potential conflict of interest.

## AUTHOR CONTRIBUTIONS

SC, CS and TJ were responsible for concept and design, analysis, interpretation of data, and final approval of the manuscript. SC and SM were responsible for data collection, preliminary analysis and drafting of the manuscript. SP and AP were responsible for laboratory consultation. SC, CS and TJ were responsible for supervising the overall conduct of the study.

## Data Availability

Further information and requests for data should be directed to and will be fulfilled by the corresponding author, Chonlaphat Sukasem. Please contact chonlaphat.suk@mahidol.ac.th.
